# Draft Genome Sequence of the Endophyte *Bacillus mycoides* Strain GM6LP Isolated from *Lolium perenne*

**DOI:** 10.1128/genomeA.00011-18

**Published:** 2018-02-01

**Authors:** Franziska Wemheuer, Jacqueline Hollensteiner, Anja Poehlein, Heiko Liesegang, Rolf Daniel, Bernd Wemheuer

**Affiliations:** a Genomic and Applied Microbiology & Göttingen Genomics Laboratory, Institute of Microbiology and Genetics, Georg-August University of Göttingen, Göttingen, Germany; b Agricultural Entomology, Department of Crop Sciences, Georg-August University of Göttingen, Göttingen, Germany

## Abstract

*Bacillus mycoides* GM6LP is an endophyte isolated from plant tissues of *Lolium perenne* L. Here, we report its draft genome sequence (6.2 Mb), which contains 96 contigs and 6,129 protein-coding genes. Knowledge about its genome will enable us to evaluate the potential use of GM6LP as a plant growth-promoting bacterium.

## GENOME ANNOUNCEMENT

Several *Bacillus* species colonize the plant endosphere (1, 2) and are frequently isolated as endophytes (3, 4). These bacteria have been found to promote plant growth and health due to their ability to induce plant systemic resistance and/or to produce antimicrobial compounds (5, 6). The genome sequence of *Bacillus mycoides* GM6LP will facilitate further studies on the potential use of these bacteria as producers of antimicrobial substances.

The strain *B. mycoides* GM6LP was isolated from surface-sterilized aerial tissues of healthy *Lolium perenne* plants. Genomic DNA was extracted using the MasterPure complete DNA purification kit (Epicentre, Madison, WI, USA). The obtained DNA was used to generate an Illumina shotgun paired-end sequencing library. Sequencing was performed employing a MiSeq system and the MiSeq reagent kit version 3 (600 cycles), as recommended by the manufacturer (Illumina, San Diego, CA, USA). Quality filtering using Trimmomatic version 0.32 (7) resulted in 2,714,322 paired-end reads. *De novo* genome assembly was performed with the SPAdes genome assembler version 3.8.0 (8). The assembly resulted in 96 contigs (>500 bp) and an average coverage of 87-fold. The assembly was validated and the read coverage determined with QualiMap version 2.1 (9).

The draft genome of GM6LP consists of 6,015,410 bp, with an overall GC content of 35.07%. Gene prediction and annotation were performed using Rapid Prokaryotic Genome Annotation (Prokka) (10). The draft genome harbored 15 rRNA genes, 69 tRNA genes, 2,358 protein-coding genes with functional predictions, and 3,773 genes coding for hypothetical proteins. Multilocus sequence typing (MLST) based on seven genes (*glp, gmk, ilvD, pta, pur, pyc*, and* tpi*) suggested by Priest et al. (11) was performed as described by Hollensteiner et al. (12). The analysis revealed that strain GM6LP belongs to the *Bacillus cereus sensu lato* group and clusters with strains of the species *Bacillus weihenstephanensis*, which has been recently reclassified as a heterotypic synonym of *Bacillus mycoides* (13).

A total of 46 potential gene clusters involved in secondary metabolite production, including 3 nonribosomal polyketide synthetase (NRPS) clusters with no or low similarity (<40%) to known gene clusters, were identified using antiSMASH 3.0.5 (14). We identified a gene cluster involved in bacteriocin production which might be beneficial for plant growth (15). A lasso peptide cluster was identified, with each gene sharing similarity to a paeninodin biosynthesis gene cluster (16). In addition, a gene cluster with 83% of the genes sharing similarity to a petrobactin biosynthesis gene cluster (17) and a gene cluster with 38% of the genes exhibiting similarity to a bacillibactin biosynthesis gene cluster known from *Bacillus subtilis* (18) were identified. Petrobactin and bacillibactin are common siderophores produced by *Bacillus* species (19). Bacterial siderophores play an important role in competition between microorganisms (20) and may promote plant growth and health by suppressing pathogenic organisms (21). In summary, *B. mycoides* strain GM6LP contains multiple gene clusters involved in promoting plant growth and health.

### Accession number(s).

The whole-genome shotgun project has been deposited at DDBJ/ENA/GenBank under the accession number MKZQ00000000. The version described here is version MKZQ01000000.
